# Asphyxia and Neonatal Respiratory Distress Syndrome Are Independent Predictors of the Non-response to Inhaled Nitric Oxide in the Newborns With PPHN

**DOI:** 10.3389/fped.2021.665830

**Published:** 2021-05-20

**Authors:** Yuwei Zhao, Lei Liang, Guanghui Liu, Hong Zheng, Liying Dai, Yan Wang, Lei Wang, Weiting Sheng

**Affiliations:** ^1^Neonatology Department, Anhui Provincial Children Hospital, Hefei, China; ^2^Pulmonary Department, Anhui Provincial Children Hospital, Hefei, China

**Keywords:** persistent pulmonary hypertension, inhaled nitric oxide, newborn, non-responder, oxygenation index

## Abstract

**Aim:** Not all the neonates respond with improvement in oxygenation following inhaled nitric oxide treatment (iNO) treatment. The aim of this study was to assess the independent risk factors associated with non-response to iNO during the 2 weeks of postnatal treatment in neonates diagnosed with persistent pulmonary hypertension (PPHN).

**Materials and Methods:** This retrospective cohort study included all newborns with PPHN who received iNO treatment for more than 24 h. Demographic, obstetric, perinatal data and clinical complications were extracted from the hospitalization records. Subjects were divided into two groups according to their response to iNO inspiration during the first 24 h of iNO treatment. No response was defined as an increase in SpO_2_ < 5% or the inability to sustain saturation levels in the first 24 h of iNO treatment. For descriptive statistics, χ^2^ and *t*-test analysis were used to compare categorical and continuous variables between the two groups. To evaluate independent risk factors of non-responsiveness to iNO treatment, binary logistic regression analysis were performed.

**Results:** A total of 75 newborns were included in the study. Sixty-two cases were in the responders group, and 13 cases were in the non-responders group. Univariate analysis showed that asphyxia, neonatal respiratory distress syndrome (NRDS), pulmonary surfactant administration, meconium aspiration syndrome (MAS), the severity of pulmonary hypertension (PH), and high-frequency oscillatory ventilation (HFOV) therapy were the high-risk factors affecting the response to iNO treatment in the newborns with PPHN. The binary logistic regression analysis indicated that asphyxia and NRDS incidence were independent predictors of non-responsiveness to iNO treatment [asphyxia: OR 4.193, 95% CI 1.104–15.927, *P* = 0.035; NRDS: OR 0.154, 95% CI 0.036–0.647, *P* = 0.011]. The patients in the non-responders group had shorter iNO inspiration followed by MV duration, supplemental oxygen and hospital stay, and higher mortality. There were no significant differences in IVH, PVL, and BPD between two groups.

**Conclusion:** In the newborns with PPHN, asphyxia and NRDS resulted as the independent risk factors of non-responsiveness to iNO therapy. Asphyxia in the newborns with PPHN is detrimental to the response to iNO treatment, while NRDS is beneficial.

## Introduction

Persistent pulmonary hypertension (PPHN) is a serious cardiopulmonary disorder in the neonatal intensive care unit (NICU) that occurs in a wide range of diseases in the neonatal period. The PPHN has been reported to occur in 1.9 of 1,000 live-born infants in the United States, 0.43–6 per 1,000 live births in the United Kingdom, and 1.2–4.6 per 1,000 live births in Asia, with mortality rates ranging from 4 to 33% (1–3). The pathophysiological manifestations in affected babies involve oxygenation decline and right ventricular dysfunction due to intrapulmonary shunts secondary to V/Q mismatch, and large extrapulmonary right to left shunts at atrial and ductal levels secondary to pulmonary hypertension (4). The PPHN-induced respiratory and circulatory failure is a life-threatening condition, which contributes to significant mortality in newborn infants with PPHN.

The general management of PPHN includes the treatment of primary diseases, ventilatory techniques for improving oxygenation, and administration of pulmonary vasodilator agents. Intravenous pulmonary vasodilators, such as prostacyclin, alprostadil, and sildenafil, have been reported in the management of neonatal PPHN (5). These pulmonary vasodilator agents would decrease both pulmonary and systemic vascular resistance; therefore, it's difficult to alleviate intrapulmonary right-to-left shunting. Besides, their efficacy and safety are still being tested.

Inhaled nitric oxide (iNO) with its fasting efficacy has been used for late preterm and term infants with persistent pulmonary hypertension and hypoxic respiratory failure for 20 years (6). iNO, which originates from vascular endothelial cells, is a pulmonary vasodilator that can improve arterial oxygenation and hemodynamic stability by reducing pulmonary vascular resistance, improving ventilation-perfusion inequalities, and reducing right-to-left intrapulmonary shunting of blood flow (7). iNO treatment has been widely used in neonates with PPHN since its first approval for use in term and near term neonates in 1999. The clinical study on neonates born after 34 weeks gestational age with persistent pulmonary hypertension reported that iNO treatment markedly improves oxygenation and decreases the requirement for extracorporeal membrane oxygenation (8). A Cochrane review on iNO for respiratory failure in neonates confirmed its effectiveness in late preterm and term newborns (9). According to these findings, iNO is an effective therapy for treating pulmonary hypertension and hypoxic pulmonary diseases. However, not all the neonates respond with improvement in oxygenation following iNO treatment. It was reported that 30–40% of neonates who received iNO treatment did not achieve optimal oxygenation (10). It still remains unclear why some newborns respond to iNO while in some, the oxygenation status does not improve after iNO therapy. Therefore, a comprehensive understanding of the effects and the outcomes of iNO may be important for the survival of infants.

At present, few studies addressed non-responsiveness to iNO treatment. The aim of this study was to assess the independent risk factors associated with non-responsiveness to iNO treatment during the 2 weeks' postnatal period in neonates diagnosed with PPHN.

## Materials and Methods

### Study Population

This retrospective cohort study included all newborns who received iNO inspiration for more than 24 h during the 2 weeks of postnatal treatment. Patients were admitted to one level-three neonatal intensive care unit in the Neonatology department of Anhui Provincial Children's Hospital between January 2016 and December 2019. Neonates who received or changed dosage of intravenous pulmonary vasodilators in the first 24 h of iNO treatment, who was given to the other changes in concomitant treatments against hypoxia, such as oxygen, distending pressure treatments, surfactant during the first 24 h of iNO treatment, as well as those with congenital malformation, complexity congenital heart defect, who died, dropped out of treatment, or were transferred within 24 h from iNO treatment, were excluded from the study.

### Diagnose of PPHN

PPHN was diagnosed based on prospective clinical and echocardiographic criteria. The clinical diagnosis was mainly based on a persistent, significant difference of ≥10 between the pre-ductal and post-ductal oxygen saturation levels and on perinatal history, physical examination, changes in oxygenation in arterial blood pressure, and chest radiographs. Echocardiographic evidence of pulmonary hypertension was defined as a systolic pulmonary artery pressure with an estimated peak higher than 35 mm Hg or more than two-thirds of the systemic systolic pressure (11). The included patients were examined by echocardiography before iNO treatment. Right ventricular systolic pressure was calculated by fluid mechanics equation: right atrial pressure (5 mmHg) + 4 (peak velocity in tricuspid regurgitation) (2). Pulmonary arterial pressure equaled with right ventricular systolic pressure. Moderate pulmonary hypertension was defined as pulmonary arterial pressure higher than 45 mmHg.

### iNO Treatment and Group Division

Mechanical ventilation mode with iNO treatment was chosen according to patients' conditions. The starting dosage of iNO was 20 ppm, which was slowly reduced by the clinically significant response. The schedule for reducing NO dosage was adopted from Soo Jung Hwang's study (12).

The included patients were divided into two groups according to their response to iNO inspiration during the first 24 h of iNO inspiration. The response was defined as an increase in percutaneous oxygen saturation (SpO_2_) of more than 5% that was sustained for the first 24 h of iNO inspiration (13). In contrast, no response was defined as an increase in SpO_2_ < 5% or the inability to sustain saturation levels in the first 24 h of iNO inspiration.

### Data Collection

Two trained research staff members collected maternal demographic, obstetric, and perinatal information. Data on detailed preterm related complications in the hospital were extracted from the hospitalization files by a dedicated pediatrician and were overseen by an attending neonatologist. The included factors were gestational age by weeks calculated from the last menstrual period and confirmed by physical examination, birth weight, small or large for gestational age, male gender, mother' age, cesarean section, steroid administration prior to delivery, and premature rupture of membranes (PROM). The factors associated with clinical characteristics before iNO inspiration were asphyxia (apgar score at 1, 5, and 10 min lower than 7), presence of neonatal respiratory distress syndrome (NRDS), pulmonary surfactant (PS) administration, meconium aspiration syndrome (MAS), pneumonia, patent ductus arteriosus (PDA), sepsis, and necrotizing enterocolitis (NEC). Data collection on the pre-iNO course included arterial blood gas analysis before iNO treatment, blood PH, PaO_2_, oxygenation index (OI = PaO_2_/FiO_2_), mechanical ventilation (MV) include conventional ventilation and high frequency oscillation ventilation (HFOV) in 24 h before iNO treatment, the initial dosage of iNO, and the time from birth to iNO initiation. The assessed outcomes were the time from iNO initiation until iNO weaning, MV duration, supplemental oxygen and hospital stay, number of patients who died, intraventricular hemorrhage (IVH), periventricular leukomalacia (PVL), and bronchopulmonary dysplasia (BPD) at corrected gestational age of 40 weeks.

### Statistical Analysis

All subsequent analyses were performed with SPSS V14. For descriptive statistics, continuous data with normal distribution were presented as mean ± standard deviation (x ± s), otherwise as a range. The *t*-test was used for comparing two groups. Categorical data were presented as the number or ratio of cases. Differences in categorical data were analyzed using the χ^2^ test or Fisher's exact test as needed.

To evaluate independent risk factors of non-responsiveness to iNO treatment, binary logistic regression analysis with significant univariate risk factors was performed. The response to iNO treatment was used as the dependent variable. The factors with *P* < 0.05 in univariate analysis were included as independent variables. The assessed outcome factors were not included. Assignment: response to iNO treatment = 1, no response to iNO treatment = 0; asphyxia = 1, no asphyxia = 0; NRDS = 1, no NRDS = 0, MAS = 1, no MAS = 0; moderate PH = 1, no moderate PH = 0; HFOV = 1, no HFOV = 0. Forward selection was used in the regression analysis. All tests were two-sided. *P* < 0.05 was considered as statistically significant.

### Ethics

All parents gave informed, written consent for the participation of children in the study. The study was approved by the institutional committee responsible for human studies.

## Results

### Perinatal and Clinical Characteristic

**Figure 1:**
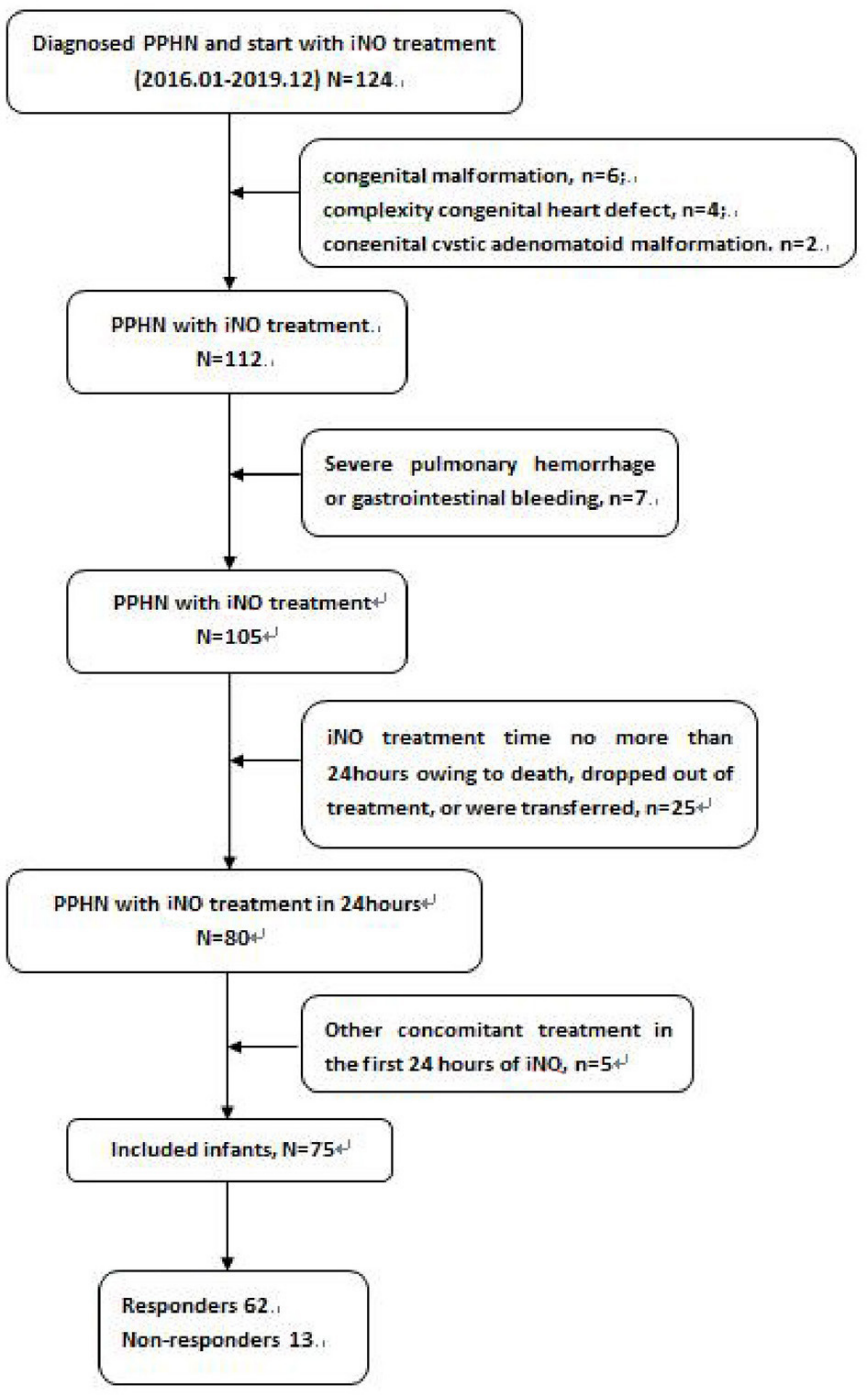
Included patients process.

Among 75 newborns included in the study, 64 were late preterm and term infants, and 11 were very preterm infants (Table 1). According to the response to iNO therapy, 62 cases were assigned to the responders' group, and 13 cases were in the non-responders group (Figure 1). There were no significant differences in perinatal factors, including gestational age, birth weight, small or large for gestational age, male gender, mother' age, cesarean section, PROM>18 h, and steroid administration prior to delivery between the two groups. Eight infants (61.5%) with asphyxia and 5 infants (38.3%) with MAS made up for higher incidence in the non-responders group (*P* = 0.012 in asphyxia; *P* = 0.015 in MAS), while 3 infants (23.1%) with RDS led to the lower incidence in non-responders group compared with responders group (*P* = 0.003). There were no significant differences in PS treatment, pneumonia, PDA, sepsis, and NEC incidence between the two groups. The newborns who did not respond to iNO treatment had more moderate PH (53.3%) compared with responders (*P* = 0.026).

**Table 1:** General information of the included patients.

**Variable n (%) or median (IQR)**	**Responders (*n* = 62)**	**Non-responders (*n* = 13)**	***x*/*t* value**	***P*-value**
**Gestational age (weeks)**				
≥34 w	38.58 ± 1.66 (*n* = 56)	38.98 ± 1.30 (*n* = 11)	−0.768	0.445
<34 w	29.66 ± 1.91 (*n* = 7)	29.40 ± 1.22 (*n* = 4)	0.240	0.816
Birth weight (g)	3,210 ± 857	3,072 ± 585	0.553	0.582
Small/large for gestational age (%)	24 (38.7%)	4 (30.8%)	0.590	0.290
Male gender (%)	50(80.6%)	11(84.6%)	0.112	1.000
Mother's age	30.44 ± 5.53	30.15 ± 6.00	0.165	0.870
Cesarean section	38 (61.3%)	7 (53.8%)	0.248	0.618
Steroid administration	5 (8.1%)	2 (15.4%)	0.680	0.409
PROM	9 (14.5%)	2 (15.4%)	0.006	1.000
Asphyxia	16 (25.8%)	8 (61.5%)	6.306	**0.012**
NRDS	42 (67.7%)	3 (23.1%)	8.933	**0.003**
PS	37 (59.7%)	6 (46.2%)	0.803	0.370
MAS	7 (11.1%)	5 (38.3%)	5.903	**0.015**
Pneumonia	20 (32.3%)	7 (53.8%)	2.174	0.140
PDA	37 (59.7%)	6 (46.2%)	0.803	0.370
Sepsis	8 (12.9%)	1 (7.7%)	0.276	1.000
NEC	4 (6.5%)	1 (7.7%)	0.027	1.000
Moderate PH	11 (17.7%)	6 (46.2%)	4.949	**0.026**

*Bold values means P < 0.05*.

### Information on iNO Treatment

Babies in both groups had similar blood pH, PaO_2_ and PaCO_2_ values (Table 2). The non-responders had lower PH and OI before iNO treatment; however, the observed difference was not statistically significant. Fifty-one infants (82.3%) in the responders group received HFOV treatment in 24 h before iNO treatment, which was significantly more compared with non-responders (*P* = 0.026). The age and dosage > 20 ppm at the initiation of iNO treatment in both groups showed no statistical significance.

**Table 2:** iNO treatment variables.

**Variable n (%) or median (IQR)**	**Responders** ** (*n* = 62)**	**Non-responders** ** (*n* = 13)**	**χ^**2**^/*t* value**	***P*-value**
**Arterial blood gas values (pre-iNO)**				
Blood PH	7.26 ± 0.10	7.19 ± 0.18	1.358	0.199
PaO_2_	62.03 ± 45.97	66.79 ± 40.72	−0.334	0.739
PaCO_2_	47.86 ± 13.81	45.88 ± 19.50	0.336	0.742
OI	23.63 ± 16.57	19.79 ± 13.57	0.756	0.452
**Ventilation mode**				
Conventional ventilation	11 (17.7%)	6 (46.2%)	4.949	**0.026**
HFOV	51 (82.3%)	7 (53.8%)		
iNO dosage > 20 ppm	12 (19.4%)	5 (38.5%)	2.238	0.135
Postnatal age at NO initiation (d)	1 (1, 2)	1 (1, 2)	−1.786	0.074

*Bold values means P < 0.05*.

### Logistic Regression Analysis

To evaluate independent risk factors of non-responsiveness to iNO treatment, the binary logistic regression analysis was performed with variables that were found to be significantly different between the groups (Table 3). These included factors such as asphyxia, diagnosis of MAS or NRDS, moderate PH, HFOV treatment with iNO. The binary logistic regression analysis indicated that asphyxia and NRDS incidence were independent predictors of non-responsiveness to iNO treatment [asphyxia: OR 4.193, 95% CI 1.104–15.927, *P* = 0.035; NRDS: OR 0.154, 95% CI 0.036–0.647, *P* = 0.011].

**Table 3:** Logistic regression analysis.

	***B***	**S.E**.	**Wald**	***P***	**OR**	**95% C.I**.
Asphyxia	1.433	0.681	4.432	0.035	4.193	1.104–15.927
NRDS	−1.874	0.734	6.513	0.011	0.154	0.036–0.647
Constant	1.783	0.682	6.834	0.009	5.946	

### Outcome of iNO Treatment

The patients in the non-responders group had shorter iNO inspiration compared with responders to iNO treatment (*P* = 0.008), which was followed by significantly shorter MV treatment, higher oxygen use, and longer duration in NICU (all *P* = 0.000). Twelve neonates (92.3%) in the non-responders group died compared to 11 neonates (17.7%) in the responders group (*P* = 0.000). There were no significant differences in IVH, PVL, and BPD between the two groups (Table 4).

**Table 4:** Outcomes of the included patients.

**Variable n (%) or quartile (IQR)**	**Responders** ** (*n* = 62)**	**Non-responders** ** (*n* = 13)**	***z*/*t* value**	***P*-value**
iNO duration, d	4 (2, 5)	1 (1, 3)	−2.652	**0.008**
MV duration	8 (6, 11)	2 (2, 4.5)	−3.978	**0.000**
Oxygen duration	16 (10, 21)	2 (2, 4)	−4.185	**0.000**
Media duration in hospital	23 (16, 29)	7 (2, 8)	−4.315	**0.000**
Died	11 (17.7%)	12 (92.3%)	28.102	**0.000**
IVH	28 (45.2%)	3 (23.1%)	2.161	0.216
PVL	2 (3.2%)	0 (0%)	0.431	1.000
BPD	2 (3.2%)	0 (0%)	0.431	1.000

*Bold values means P < 0.05*.

## Discussion

iNO inspiration is an effective therapy for the treatment of pulmonary hypertension. Nevertheless, 30–40% of neonates who received iNO treatment did not experience improvement in oxygenation. At present, few studies reported on the effects and the outcome of iNO treatment. This study aimed to assess the independent risk factors associated with non-response to iNO treatment during the two postnatal weeks in neonates diagnosed with PPHN. Univariate analysis showed asphyxia, NRDS, MAS, the severity of PH, and HFOV therapy were the high-risk factors affecting the response to iNO treatment in the newborns with PPHN. The binary logistic regression analysis indicated that asphyxia and NRDS incidence were independent predictors of non-responsiveness to iNO treatment. Asphyxia in the newborns with PPHN is detrimental to the response to iNO treatment, while NRDS is beneficial. Besides, the patients in the non-responders group had shorter iNO inspiration, followed by MV duration, supplemental oxygen and hospital stay, and higher mortality.

Persistent pulmonary hypertension of the newborn (PPHN) is a serious syndrome characterized by the elevated pulmonary vascular resistance (PVR) at birth. iNO treatment has been widely used in late preterm and term infants with persistent pulmonary hypertension and hypoxic respiratory failure. Nitric oxide originates from the endothelial cells and causes pulmonary vasodilation through the generation of cyclic guanosine monophosphate (cGMP). Unlike some pharmacologic agents, iNO, which is bound by hemoglobin in the circulation, has minimal systemic vasodilator effect. iNO causes an improvement in arterial oxygenation and hemodynamic stability by reducing pulmonary vascular resistance, improving ventilation-perfusion inequalities, and reducing right-to-left intrapulmonary shunting of blood flow (14). Thus, far, iNO remains the only pulmonary vasodilator therapy for neonates with PPHN approved by the United States Food and Drug Administration (FDA). While iNO therapy had been widely used in term and near-term neonates, the response to iNO remains suboptimal. A Cochrane systematic review of iNO therapy for respiratory failure revealed that almost half of near-term and term infants with hypoxic respiratory failure had suboptimal improvement in oxygenation after receiving 30–60 min iNO (9). Morel et al. showed a high effective rate with 31 out of 44 newborns (70%) responding to iNO treatment within 6 h (15). Nelin et al. studied 126 neonates receiving iNO treatment, among whom 106 had ≥10% increase in PaO_2_ and/or ≥10% decrease in OI, which suggested an initial oxygenation response to iNO treatment (8).

The present study investigated iNO treatment response and associated clinical characteristics and factors in neonates with PPHN, thus offering vital information for identifying critical newborns likely to non-respond to iNO therapy. Due to the possibility of a rebound pulmonary hypertension with suppression of endogenous nitric oxide production, responders in this study were defined as patients who had an increase in FiO_2_ more than 5% that was sustained in the first 24 h of iNO inspiration. In our tertiary neonatal center, over a period of 5 years, ~13.33% of neonates with PPHN did not respond to iNO therapy within 24 h. Our current results were similar to the study by Nelin et al. who examined 106 neonates receiving iNO treatment with improved oxygenation, among whom 66% responded within <= 30 min, 12% within <= 1 h, 8% within <= 24 h, and 14% after 24 h. They reported that 18.87% of neonates did not have an initial oxygenation response to iNO treatment (8).

Most previous studies discussed the dosage and efficacy of iNO therapy; however, specific studies on non-responders to iNO therapy are rare. It is still unclear why some infants show no significant response to iNO. This study assessed the specific information on the perinatal and clinical period to identify the factors associated with lack of improvement in oxygenation after 24 h of iNO treatment in newborns. The results of our univariate analysis showed that newborns who did not experience improvement in oxygenation after 24 h of iNO therapy had a higher occurrence of asphyxia and MAS, fewer NRDS, worse pulmonary artery hypertension, and less HFOV therapy. However, binary logistic regression analysis that included these variables revealed asphyxia and NRDS as the independent risk factors of non-responsiveness to iNO treatment in newborns with PPHN. In this study, the newborns with PPHN who showed no response to iNO treatment had a 4.193-fold increased risk of asphyxia and a 85.6 percentage decreased risk of NRDS. It suggested asphyxia in newborns with PPHN is detrimental for the response to iNO treatment, while NRDS is beneficial.

Newborns with asphyxia at birth are always at high risk of pulmonary disease, particularly PPHN (16). Hypoxic pulmonary vasoconstriction is maintained in the fetus due to the low resting arteriolar and alveolar oxygen tension. At birth, following the initiation of respiration, alveolar oxygen tension and ventilation increase; however, asphyxia at birth can interfere with the pulmonary transition during birth, thus increasing the risk for severe pulmonary hypertension. Perinatal asphyxia induces a marked reduction in enzyme nitric oxide synthase (NOS), which requires the presence of oxygen for the production of endogenous NO (17). Besides, a key factor for determining the bioavailability of NO in a tissue is the local concentration of superoxide anions. Asphyxia induced accumulation of oxidative stress include superoxide anions that may potentially blunt the effect of NO (18). A previous study on the term and late-term infants reported significantly lower apgar score at 1 and 5 min of life in the non-responders group, while the association was not significant in the regression model (15). This study included asphyxial infants with apgar score at 1, 5, and 10 min lower than 7 who showed no response to iNO treatment. However, one study on preterm neonates at GA < 34 weeks did not report severe asphyxia in non-responders (19).

In the present study, neonates with NRDS had a better response to iNO treatment, significantly different from neonates without NRDS. More babies with pneumonia and MAS failed to respond to iNO treatment; still, without statistical significance, which was consistent with the studies by Hwang et al. (12) and Morel et al. (15). Both of these studies reported that more of neonates with RDS responded well to iNO treatment; the results were statistically significant. Hypoxia in RDS is a result of primary pulmonary surfactant deficiency. It is considered that hypoxia in the acute stage of RDS may alter the physiological function of NO (12). Besides, most newborns with NRDS get pulmonary surfactant administration that could contribute to improve oxygenation if administered before iNO (20). Further research on the specific mechanism between RDS and iNO is still needed.

The present study has some limitations. First, we did not specify unified criteria for lack of response to iNO treatment; we used sustained and steady SpO_2_ to assess the response to iNO. Second, we had a relatively small sample size, and restricted patient population, thus larger multicenter trials should are needed to validate our findings.

## Conclusion

This study demonstrated that asphyxia and NRDS were the independent risk factors for non-response to iNO therapy in newborns with PPHN. Asphyxia in the newborns with PPHN is detrimental to iNO treatment response, while NRDS is beneficial. This study could assist clinicians in assessing response to iNO and determine optimal timing for iNO weaning.

## Data Availability Statement

The original contributions presented in the study are included in the article/supplementary material, further inquiries can be directed to the corresponding author/s.

## Ethics Statement

Written informed consent was obtained from the individual(s), and minor(s)' legal guardian/next of kin, for the publication of any potentially identifiable images or data included in this article.

## Author Contributions

YZ collect the data and wrote the manuscript. LL analyzed the data. GL funded the study. LD and HZ reviewed and edited the manuscript. YW, LW, and WS collect the data. All authors read and approved the manuscript.

## Conflict of Interest

The authors declare that the research was conducted in the absence of any commercial or financial relationships that could be construed as a potential conflict of interest.
